# The School Garden: A Social and Emotional Place

**DOI:** 10.3389/fpsyg.2021.567720

**Published:** 2021-04-22

**Authors:** Susan Pollin, Carolin Retzlaff-Fürst

**Affiliations:** Department of Didactics in Biology, University of Rostock, Rostock, Germany

**Keywords:** school garden, social interactions, communication, cooperation, emotions

## Abstract

School gardens are part of many schools. Especially in primary schools, but also in secondary schools, they are used as a learning space and experience space for the pupils. Their importance for the development of cognitive and emotional-affective abilities of pupils is empirically well proven. It is also empirically well proven that exposure to nature has an influence on the prosocial behavior of children and adults. However, there is a lack of studies investigating the effect of the stay in the school garden on the social behavior of pupils in secondary class. To investigate whether a school garden is a good environment for social learning, a self-report study and standardized observations with sixth-grade pupils were carried out. Thus, the socially competent behavior of the pupils (communication and cooperation) and their emotions could be analyzed. In order to provide emotional access to the scientific content of biology lessons and to strengthen social learning, each pupil was responsible for their own plant and the group bed over a period of 10 weeks. The design of the lessons followed the principles of basic needs—competence, autonomy, and relatedness—of the Self Determination Theory. The observations were made during a 90-min class, in the school garden as well in the classroom. The 31 girls and 22 boys, aged 11–12 years, changed weekly between the garden and the classroom. Over 150 observations were made in the school garden (82) and in the classroom (68). In summary, pupils showed more socially competent behavior in school garden lessons than in classroom lessons. The school garden lessons, designed according to the basic needs, seem to create favorable incentives for social learning. Due to frequent social interactions, it can be assumed that learning activities in school gardens can promote emotional and social competence.

## Introduction

Everyone has the need to develop themselves and to have their own experiences in interaction with others. However, there must be opportunities to develop personal skills: cognitive, psychomotor, emotional, and social skills. A natural space, such as the school garden, offers these possibilities independently of age and class level. In the school context, natural spaces on school grounds provide a personal encounter with nature and living organisms such as plants, animals, and fungi. These interactions with nature and the possibility to become active in the garden are combined with social and emotional skills. This is connected with the development of pupils' attitudes, values, beliefs, and self-perceptions.

Studies worldwide examine the psychological and physiological effects of contact with nature in the form of gardens, parks, green spaces, and forests on the human organism (Brown et al., 2013; Haluza et al., 2014; Dadvand et al., 2016; Shanahan et al., 2016; Cox et al., 2017). There is evidence for a positive relationship between access to green or natural environments and people's social behavior (Taylor et al., 2001; Maas et al., 2009; Faber Taylor and Kuo, 2011; Rash et al., 2011; Putra et al., 2020). School gardens are also places of encounter with each other (Dyment and Bell, 2008; Malberg Dyg and Wistoft, 2018) in nature. Social skills learning concerns communication and cooperation, the ability to relate to others, and teamwork. The development of prosocial behavior through staying in nature is well-documented for children and adults (Carney et al., 2012; Carrus et al., 2017; Putra et al., 2020). These studies refer to the effect of staying in public urban spaces or in the school environment on adults and children—especially in primary schools. The few empirical studies with children and adolescents predominantly refer to spending time in gardens. Students who engage in gardening have better social relationships. Gardening can also lead to better connections between neighbors in the family environment (Wells et al., 2014).

Personal encounters with nature and with natural organisms are usually associated with positive emotions and connotations. For adult individuals, it has been demonstrated that exercise in the natural environment, as opposed to an urban environment, can lead to positive emotional states (Berman et al., 2008; Mayer et al., 2009; Johansson et al., 2011). If the natural environment is considered a place of leisure, it can lead to restful or relaxing experiences and self-reflection (Kaplan and Kaplan, 1989; Kaplan, 1995). For adults, it was found that green spaces that are perceived as beautiful have a positive influence on prosocial behavior (Zhang et al., 2014). Girls and boys seem to benefit differently from green spaces. Public parks are important for boys, while girls benefit more from playgrounds and recreational areas (Richardson et al., 2017). However, encounter with nature can also evoke negative emotions and connotations (Bixler and Floyd, 1997; Sugiyama et al., 2021). For example, negative emotions are often associated with encounters with invertebrates such as spiders or snails (Wilson, 1987; Retzlaff-Fürst, 2005; Wagler and Wagler, 2018).

Our exploratory study examined the extent to which biology lessons in school gardens affect the development of social and emotional behavior. Our target group consists of pupils of the sixth grade.

The first aim of the study is to record the emotions experienced by the pupils in the classroom and in the school garden. Emotions are an integral part of well-being. According to Becker (2006), the pupils' satisfaction of needs leads to an increased sense of well-being. Positive and negative emotions develop depending on the situation. The first question is: What emotions were perceived by the pupils during lessons in the classroom and in the school garden?

The second aim of the study refers to social competence. Social interactions take place in every situation and at every place. A high number of social interactions, in the sense of “socially competent behavior,” suggest a high level of social learning and a high degree of social inclusion. The second question is: Which social interactions can be observed in terms of “socially competent behavior” in direct verbal communication and cooperation in the classroom and in the school garden lessons?

## Materials and Methods

### Design

The development of a good social learning environment in the school garden was studied applying the design-based research method (Akker et al., 2013). Therefore, pupils of the sixth grade, between the ages of 12 and 13, of a cooperating comprehensive school were taught in the classroom and in the school garden. The location of the science lessons changed weekly from the classroom to the school garden. Learning modalities, like materials and organization, for the school garden were tested and pupils' emotions and social interactions were investigated exploratively. After the pilot phase in the year 2015, the first field study with five classes (study 1 with 124 pupils, 59 boys and 65 girls), divided into half classes, followed in 2016. In the next year 2017, a repeat study (study 2 with 53 pupils, 22 boys and 31 girls) with two classes was conducted. In every study, an analysis phase follows a trial phase. The data published here come from the repeat study (study 2, *n* = 53). The content of “Plants and Soil” was therefore practically and theoretically taught in the school garden and in the classroom. The independent gardening was carried out in groups, with particular emphasis on contact with nature and the satisfaction of psychological needs—basic needs according to the self-determination theory (Deci and Ryan, 2004).

### Social Interaction and Emotions

Assuming that people are born and live in a social world, interpersonal interactions are essential for their personal development and life in society. Social behavior is learned from people through appropriate interpersonal situations (socialization), e.g., also when watching other people or when interacting with people. The school institution has a significant role to play in this. According to educational guidelines, social competence is promoted among schoolchildren. In the course of school socialization, socially competent behavior is promoted and values and norms are conveyed. The term “social competence” is used at the level of education policy and school education. An executive definition of social competence is “… the availability and application of cognitive, emotional and motor behaviors that lead to certain social situations in a long-term favorable relationship of positive and negative consequences for the agent” (Hinsch and Pfingsten, 2015, p. 18). Social competence defined in short is “effectiveness in social interaction” (Rose-Krasnor, 1997). It is a multidimensional concept closely related to the terms “communication” and “cooperation” (Reichenbach, 2014). There is a variety of related terms, such as social action (of sociology), social–emotional behavior, social skills, and social learning (of pedagogy), which are used depending on the relevant context and field of science. The concept of social interactions and the concept of socially competent behavior, according to Kanning (2002), are used for this work. He describes a subarea as social orientation, associated with prosociality and listening, and “socially competent behavior” as an evaluative term that concerns the person, the situation, and the local context. Accordingly, socially competent behavior is the “behavior of a person that contributes to the achievement of one's own goals in a specific situation, while at the same time maintaining the social acceptance of the behavior” (Kanning, 2009, p. 15). In this work, we especially looked at the school and the everyday school situation as context, the pupils as the acting persons. Emotions have important functions, on biological levels (e.g., body base and emotional quality), on psychological levels (e.g., expression and development), and on social levels (e.g., communication) (Hülshoff, 2012). According to the multidimensional perspective, emotions influence several behavior systems that shape the subjective emotional experience component (Eder and Brosch, 2017) and are composed of different components: the physiological component, such as changes in blood pressure, skin conductivity, and respiratory rate; expressive components, such as voice, facial expressions, and posture; cognitive components, such as the accessibility of information in the memory; and motivational components, such as generating motivation and willingness to act. There is no consensus among psychologists working on emotions about what exactly emotions are. To use a “working definition” by (Eder and Brosch, 2017, p. 188): “An emotion is an affective reaction aimed at a specific object, which is accompanied by temporary changes in experience and behavior.” According to Eder and Brosch (2017), the following characteristics of emotions are affectivity (emotional character): emotional experience, for example, fear; intentionality (object orientation): reference to the object can be established; temporal dynamics; and limited duration. After evolutionary (phylogenetic) development processes, Ekman (1992) argues that there are specific emotions that represent independent systems—basic emotions. In his first research, he particularly referred to facial expressions from different cultures. The majority of cultures (over five cultures) show that facial expressions of corresponding emotions are universal. This was also shown in other cultural groups (Izard and Murakami, 1994; Ekman et al., 2011). According to Ekman and Friesen (1971), happiness, fear, anger, sadness, surprise, and disgust can also be recognized from facial expressions (photographs). These emotions are, therefore, discrete, which meet the following characteristics (Ekman, 1992): distinctive universal signals, presence in other primates, distinctive physiology, distinctive universals in antecedent events, coherence among emotional response, quick onset, brief duration, automatic appraisal, and unbidden occurrence. The emotion psychologist Paul Ekman is not the only representative of the theory of basic emotions. Turner (2007) compared 20 different lists of primary emotions. According to Izard, in addition to Ekman's basic emotions, embarrassment, excitement, interest, and guilt are to be named (Izard and Murakami, 1994). It is assumed that by mixing up basic emotions, new types of emotions appear—the secondary emotions.

### Teaching in the School Garden and in the Classroom

**Figure 1:**
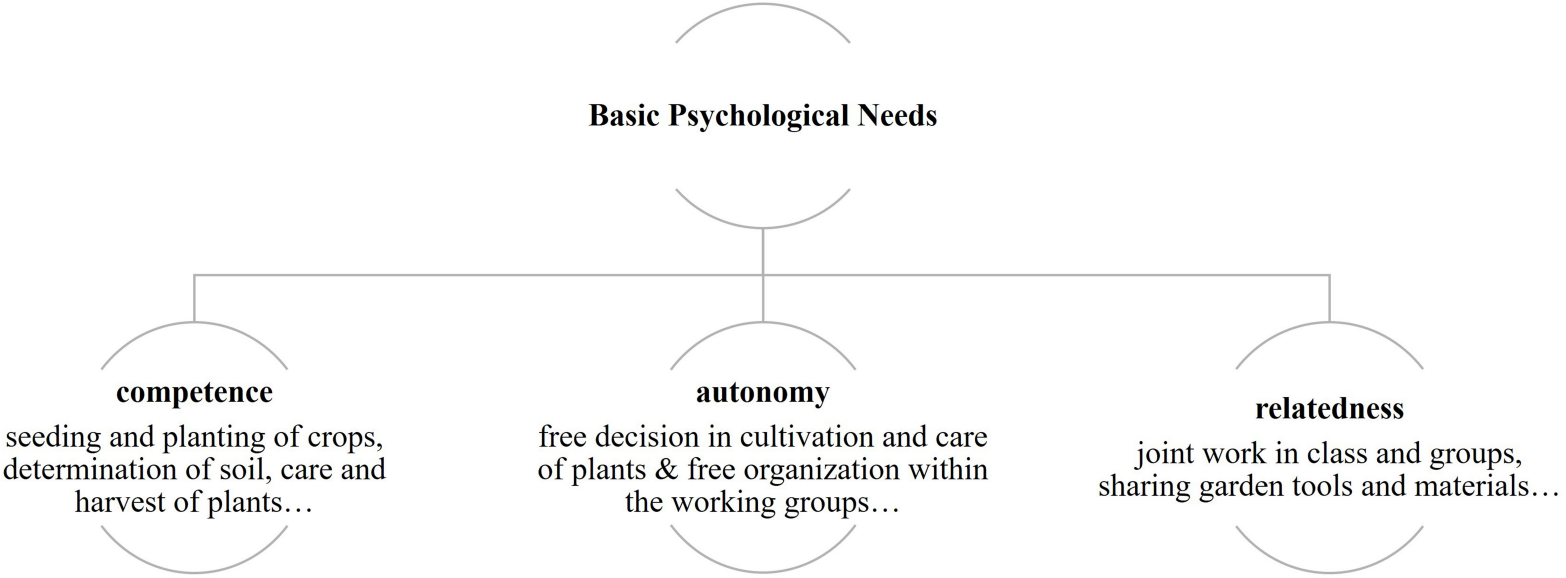
Realization of basic needs—Self Determination Theory (SDT).

In science lessons, creatures are crucial for gaining knowledge; they stand for an original encounter, realism, and illustrative material (Spörhase-Eichmann and Ruppert, 2010). The school garden lessons give plenty of opportunity for primary experiences, through the cultivation of and contact with plants as well as through accidental contact with animals, for example with soil organisms such as earthworms, spiders, and woodlice. Further importance of learning in the garden lies in promoting the interest of the pupils. It is known that girls and boys in the age of 15 show little interest in the topics of plants and plant growth (Schreiner, 2006, 2012). Prokop et al. (2007) report that science lessons are becoming more and more theoretical. The interest of the pupils can be encouraged through the possibility to design and maintain their own garden bed independently and to harvest the plants (Krapp, 2002). It is generally known that teaching with living beings promotes motivation to learn. Responsibility for their own plant bed can promote motivation and a caring attitude. Under these assumptions, all five classes of the sixth grade included in the study were taught in the school garden and in the classroom under the same conditions—following the normal science curriculum, on the topics of “soil and plants.” Therefore, during a period of 10 weeks, the pupils (31 girls and 22 boys) changed weekly between the garden and classroom. The science lessons in the classroom were held as usual by the subject teachers. As an organizer and moderator, they lead the class in the classic sense. The science lessons in the school garden were held under the conditions of basic needs (Deci and Ryan, 2004)—competence, autonomy, and relatedness—of the Self Determination Theory (SDT). The fulfilment of basic needs is seen as the basis for good teaching (Figure 1), which focuses on motivation and well-being. The basic psychological needs are the following: competence—to experience oneself as a subject able to act, to fulfil tasks on one's own; autonomy—to experience the achievement of goals by one's own, self-determined action; and relatedness—feeling connected and accepted by a group.

For the teaching design, this means that the pupil groups were given the responsibility for the bed, they could manage the gardening independently and on their own. Tasks in the garden, besides bed care, were documented regarding plant growth, snail observations, soil tests, and plant identification. During the gardening activity, the teacher was in the social role of advisor and time watchman—the teacher introduced the garden lesson, checked the process, and ended the lesson.

### Instruments and Implementation

**Figure 2:**
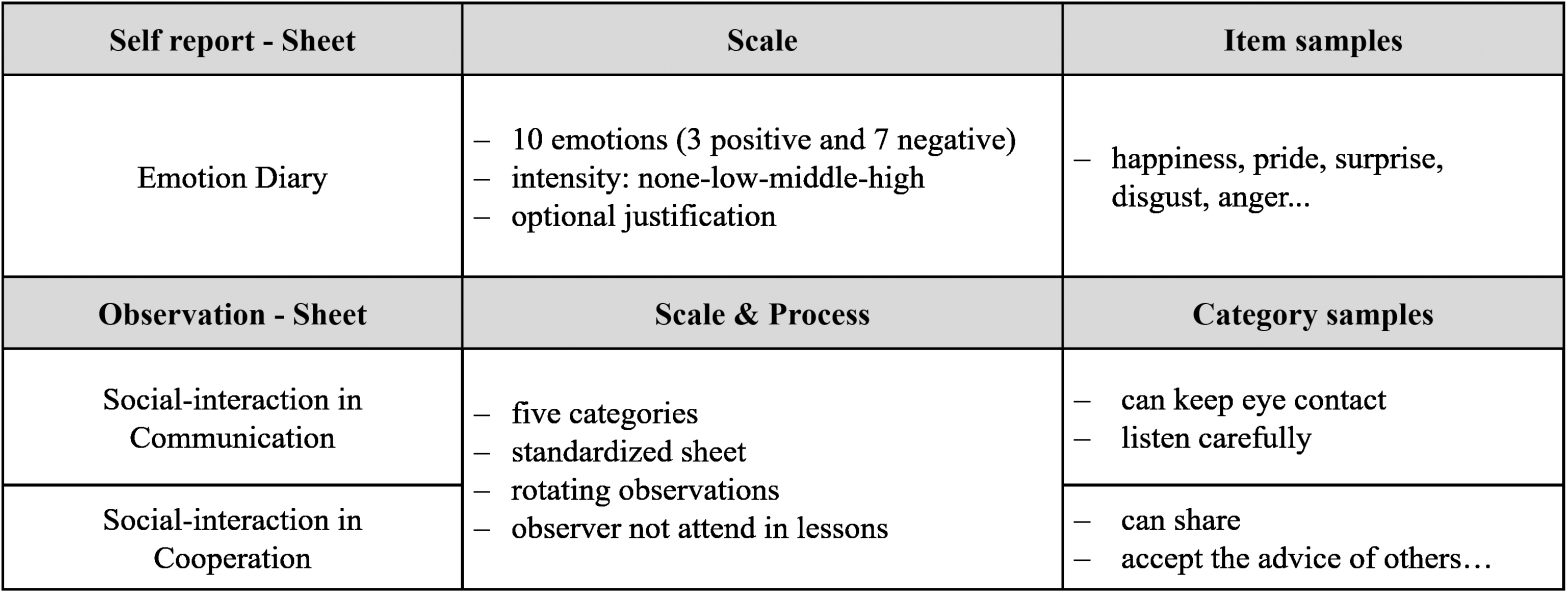
Overview of the instruments.

For data acquisition, a self-report study and an observational study were carried out during a period of 10 weeks of lessons. The instruments were developed for the purpose of this study; Figure 2 gives an overview of the instruments. The self-report “emotion diary” was distributed after each lesson in the classroom and after each lesson in the school garden. It is a kind of checklist (in the Annex), a robust test and not further validated. For this paper-and-pencil test, the pupils needed about 15 min to fill out. In this way, all sixth-grade pupils were asked weekly about their emotions during the lessons. The list of emotions was compiled on the basis of the emotion theories of basic emotion by Ekman (1992) and Izard and Murakami (1994). However, the communicative validation showed that a sufficient differentiation of emotions (basic emotions) cannot be assumed for sixth-grade students. Therefore, the emotions that apply similarly were summarized such as rage and anger, wonder and surprise, and fear and anxiety brought together in one answer category each. According to the general interpretation of Cronbach's alpha values (Cronbach, 1951), the emotion diary can be classified as acceptable. In the piloting of the emotion diary, the test run showed Cronbach's α 760 (*n* = 23) for the positive emotions and Cronbach's α 790 (*n* = 22) for the negative emotions. The pupils' acceptance and willingness to provide information were ensured through informative introductory discussions and exercises on the test instruments.

For the standardized observation, seven observers were trained for the documentation of social interaction. The observations were made at two to three 10-min intervals with a break of 5 min in between. The focus of this structured observation was on prosocial communication and cooperation—the counting of the frequency of “socially competent behavior” was trained in simulation of gardenwork. The observers rotated from one group of pupils to the next, and a so-called lump sample was carried out (Blanz, 2015). In the school garden, the pupils were equipped with colored shirts (bibs) and different symbols for group membership. An average of three observation protocols per observer was kept during one lesson.

The observation sheet (in the Annex) was developed after theoretical work on the topic of social competence. Five specific indicators of communication and cooperation were developed for the categories of prosociality, with the aim of mapping the respective categories neither too extensively nor too imprecisely. Dimensions such as empathy, conflict behavior, or extraversion were discarded for reasons of expediency (with regard to economy, practicability, and susceptibility to distortion of perception). The training of the observers included the repetition of the quality criteria, the presentation of the study, familiarization with the observation sheet, and the communication of frequent observation errors (distortions), as well as practical observation runs. Exercises in the garden with a final evaluation were essential in order to be able to distinguish the items from one another. The piloting showed an interobserver agreement of 80%, which is to be assessed as acceptable (Wellenreuther, 2000).

Data analysis of the recorded emotions and observed social interactions was done using SPSS (from IBM SPSS Statistics, version 25), a common statistical program. The paired samples of each pupil were used for descriptive statistics of the emotions. The differences were checked for statistical significance on the basis of ordinal and nominally scaled data using the Mann–Whitney *U*-test (non-parametric test). The Excel software program was used to calculate the mean of social interactions. The calculated effect sizes *d* are interpreted according to Cohen (1988, 1992): small effect from 0.2, medium effect from 0.5, and large effect from 0.8.

## Results

### Emotions in the School Garden vs. Classroom

**Figure 3:**
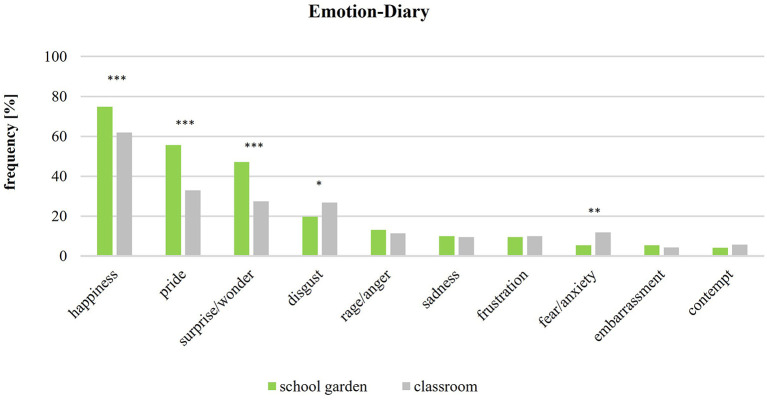
Emotions in school garden and in classroom (school garden *n* = 223; classroom *n* = 215). Significance p different levels (*p < 0.05; **p < 0.01; ***p < 0.001).

The emotions of the pupils (*n* = 53, 22 boys and 31 girls) were measured by an emotion diary. The results show which emotions were perceived by the pupils during the school gardening (colored green) and in the classroom (colored gray). Moreover, they also show whether the emotions were related to what is going on and to what intensity positive and negative emotions were perceived in the school garden class and in the classroom class. All in all, the data were recorded in 223 sheets at the school gardening and in 215 sheets at the classroom teaching. Figure 3 shows that 5 out of 10 emotions were perceived significantly (*p* < 0.001) more often—happiness, pride, surprise/wonder more often in the school garden and disgust, fear/anxiety more often in the classroom.

The computed differences (Mann–Whitney *p* < 0.001) show that in the school garden the emotions happiness 12%, pride 22%, and surprise/wonder 19% were perceived more often than in the classroom (according to Cohen 1988, with small effects *r* = 0.1 up to *r* = 0.2). On the other hand, emotions of disgust were reported 7% more often and fear/anxiety 6% more often after the teaching in the classroom—with small effects *r* = 0.1. Overall, emotions in the school garden are stated 43% more often than in the classroom. On the other side, the emotions rage/anger, sadness, frustration, embarrassment, and contempt do not show any significant difference and effects in frequency (Mann–Whitney test).

**Figure 4:**
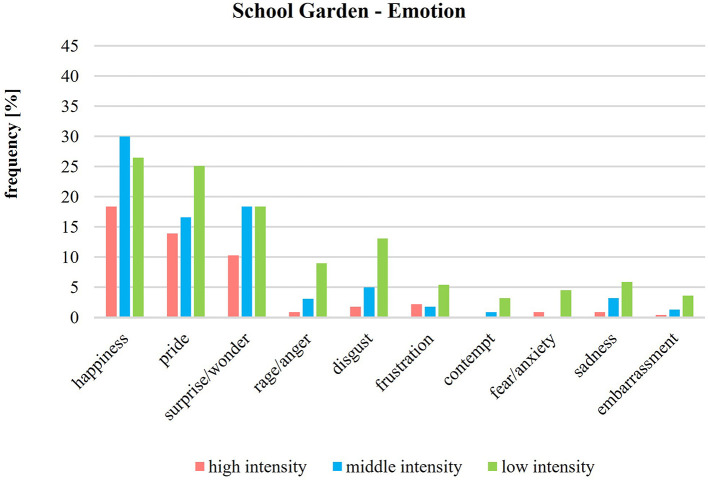
Intensity of emotions during school garden lessons (*n* = 223).

**Figure 5:**
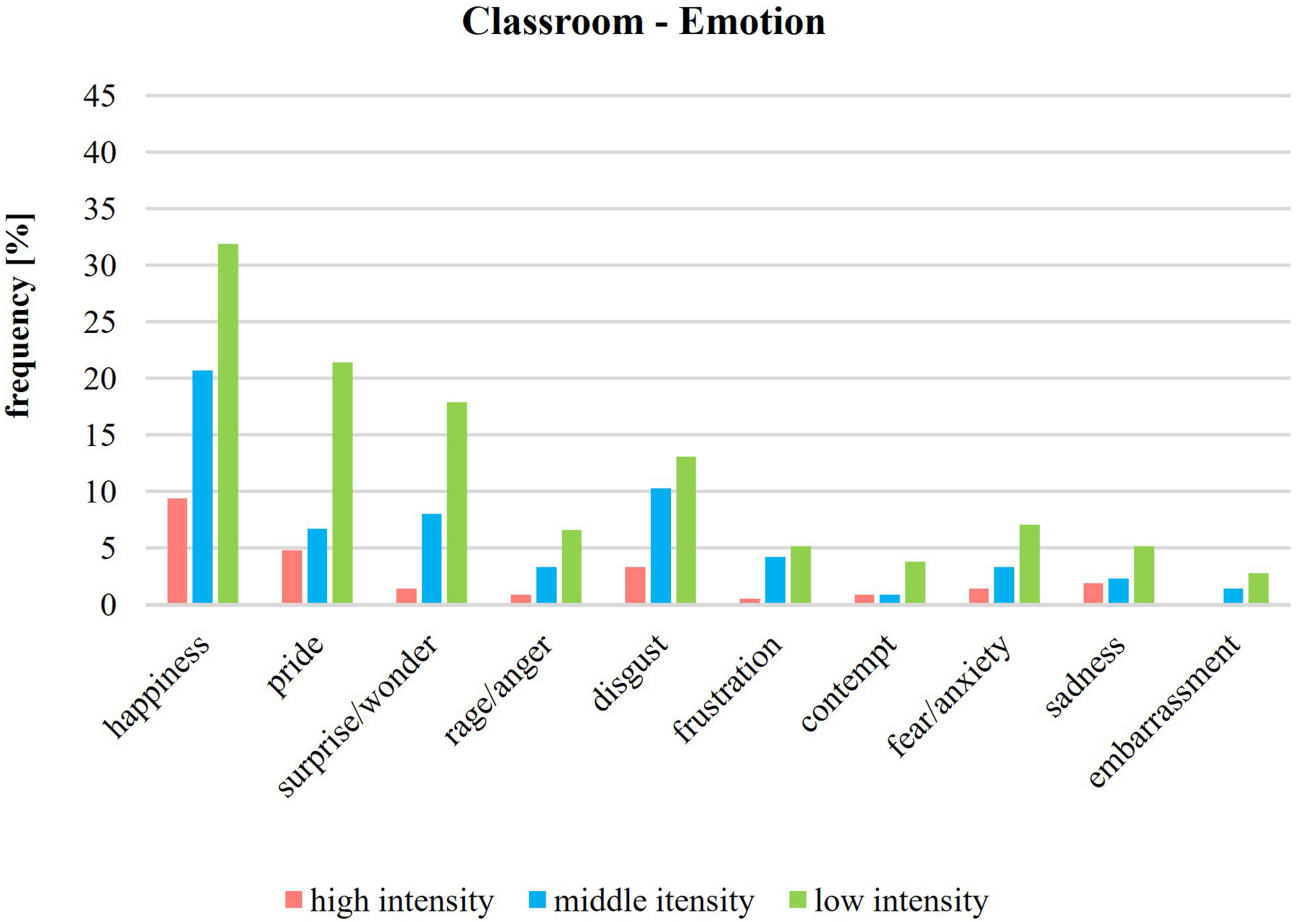
Intensity of emotions during classroom lessons (*n* = 215).

Figure 4 presents the emotions in the school garden according to their intensity levels. Regarding positive and negative emotions, the frequencies of the intensity levels (low, middle, high) show that in the school garden, positive and negative emotions are often perceived at low or middle intensities. However, positive emotions in the school garden are often intensively perceived—in contrast to the negative emotions. The emotions of happiness, pride, and surprise/wonder make up 85% of the total high intensity. The intensity of emotions in the classroom (Figure 5) is generally often low. Happiness is the only emotion that is over 8% in average.

**Figure 6:**
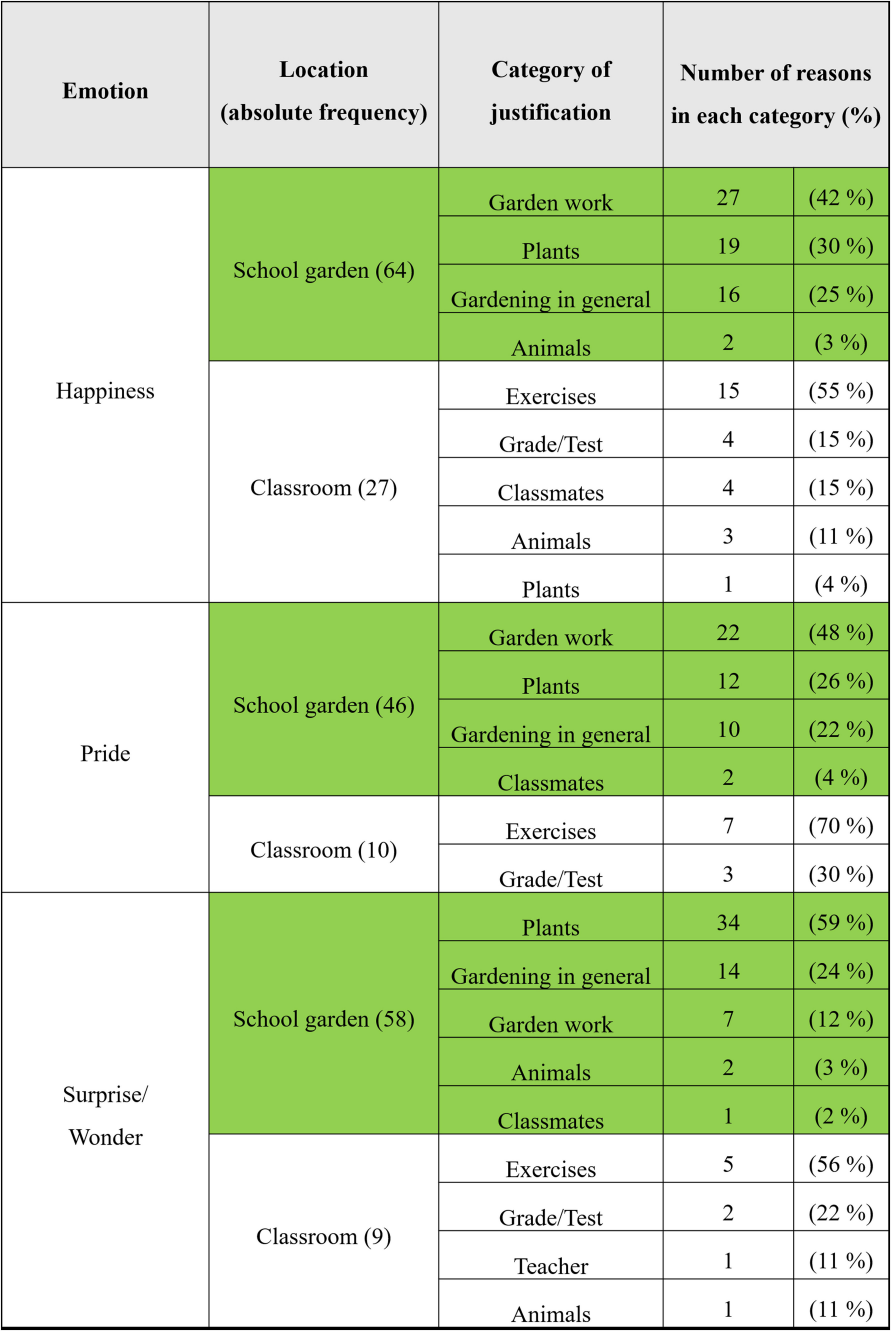
Reasons of positive emotions (school garden *n* = 223; classroom *n* = 215).

**Figure 7:**
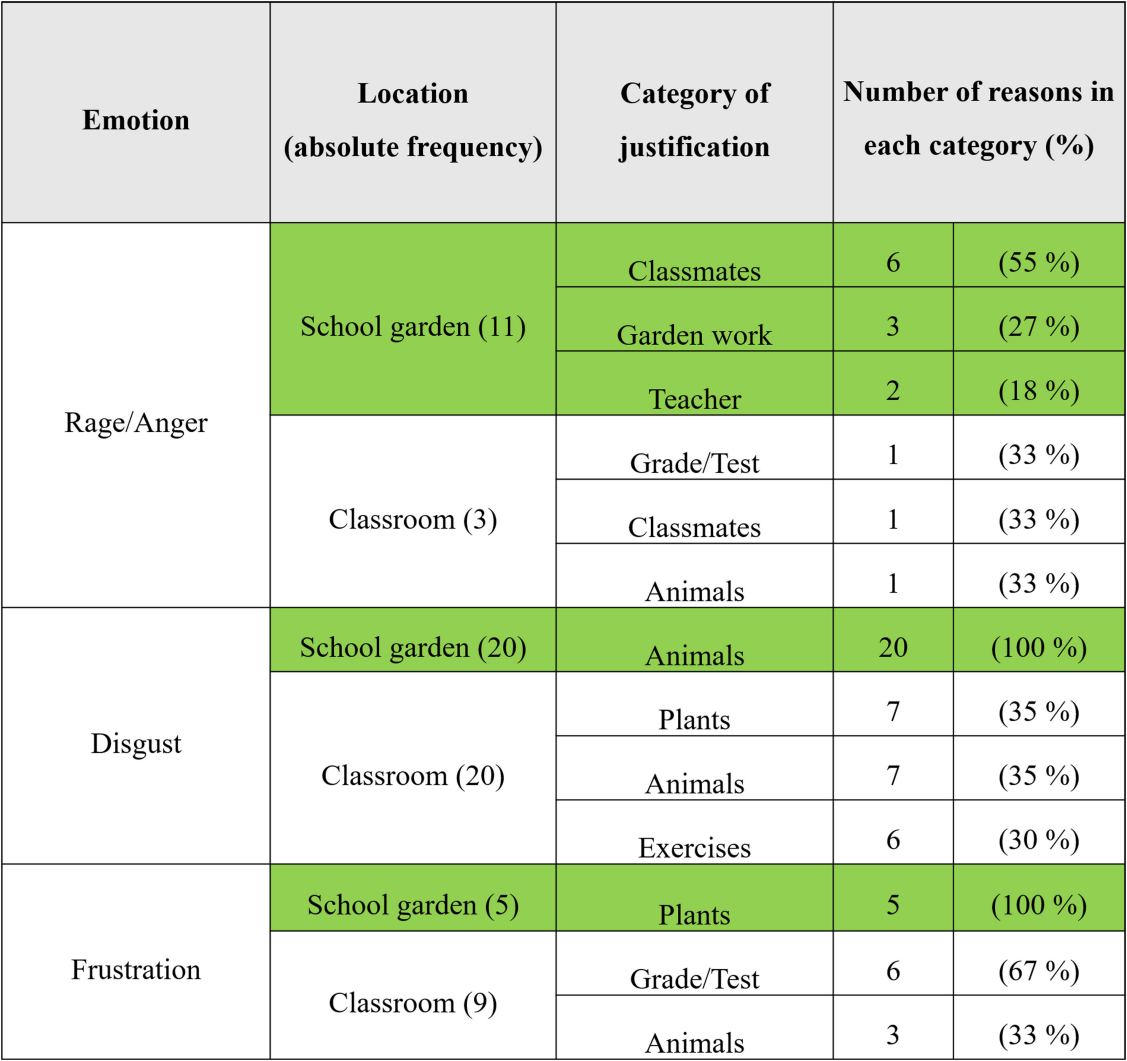
Reasons of negative emotions (school garden *n* = 223; classroom *n* = 215).

In the emotion diary, pupils informally and freely give reasons for the emotions perceived by them in brief statements about the situation. Figure 6 outlines that in the school garden joy/happiness (64), pride (46), and surprise (58) are often justified by statements about gardening, plants, and animals. On the other hand, in Figure 7, in the classroom, the pupil's comments are less about plants and animals (4) and more often about lessons in general (26) and tests or presentations (9).

Reasons for the emotions reported that pupils have noted in the emotion diary after the school gardening are as follows: happiness: “that we did something in the bed,” “found earthworm,” “had cool flowers,” “because the plants have grown,” and “we are in the garden”; pride: “bed looks good,” “on the pumpkins,” “my flowers,” “everything has grown well,” “on our harvest,” and “that we did it”; and surprise and wonder: “because everything was fully grown again,” “that pumpkins can get so big,” “worms, snails, millipedes,” “the bed was so big,” and “that we work so well in a team.”

For the negative emotions, the reasons are as follows: rage and anger: “because I worked alone with a mate,” “when Biggi was annoying all the time,” “neighbor's,” and “because of the bed”; disgust: “because of a mosquito,” “spiders and beetles,” “disgusting spider,” “soil animals,” and “strange yellow-black tabby insect in the radishes”; and frustration: “sunflowers” and “because carrots unfortunately didn't really grow.”

### Social Interactions in the School Garden vs. Classroom

**Figure 8:**
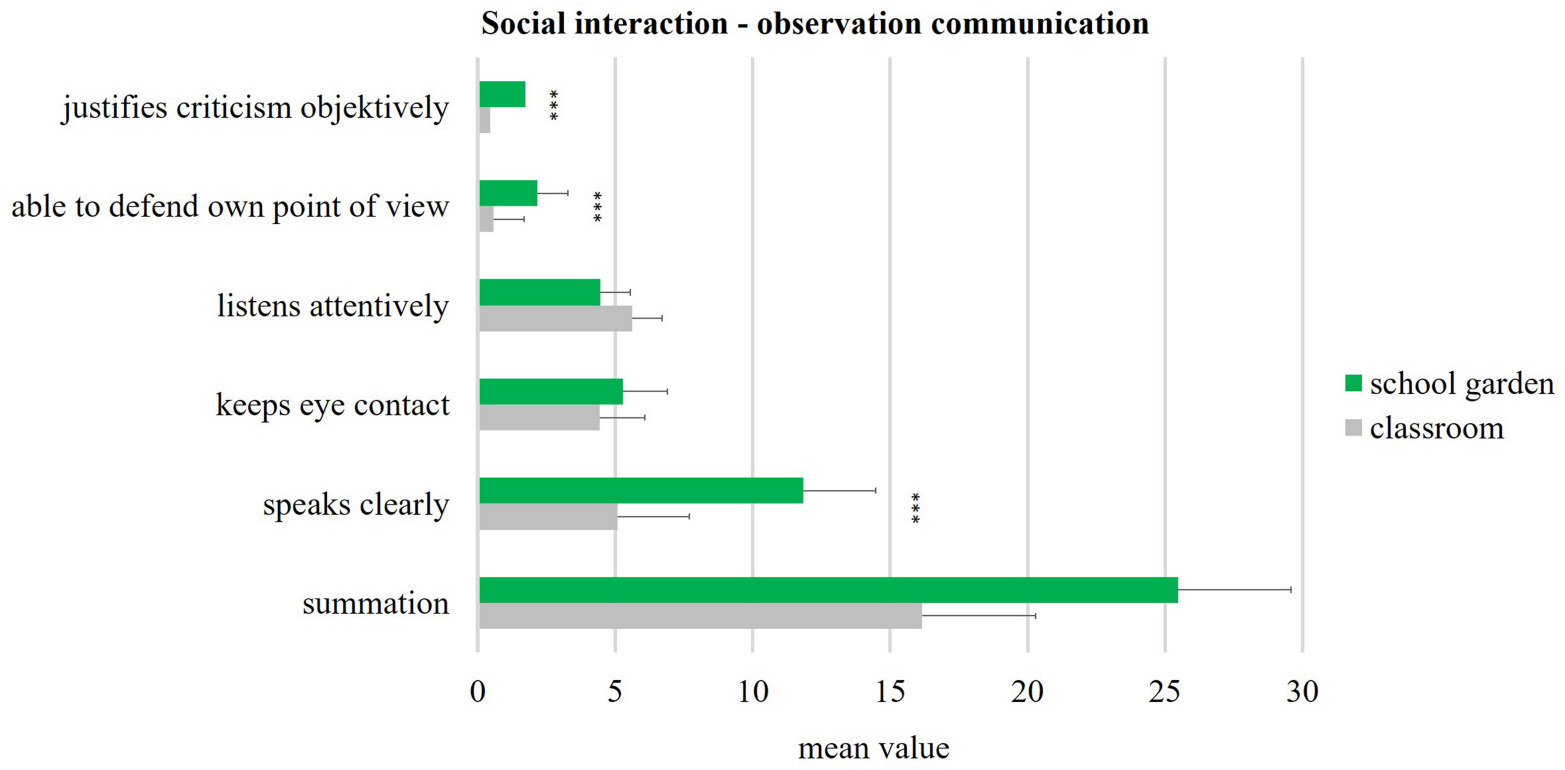
Social interaction - communication (school garden *n* = 82, classroom *n* = 68). Significance p different levels (*p < 0.05; **p < 0.01; ***p < 0.001).

**Figure 9:**
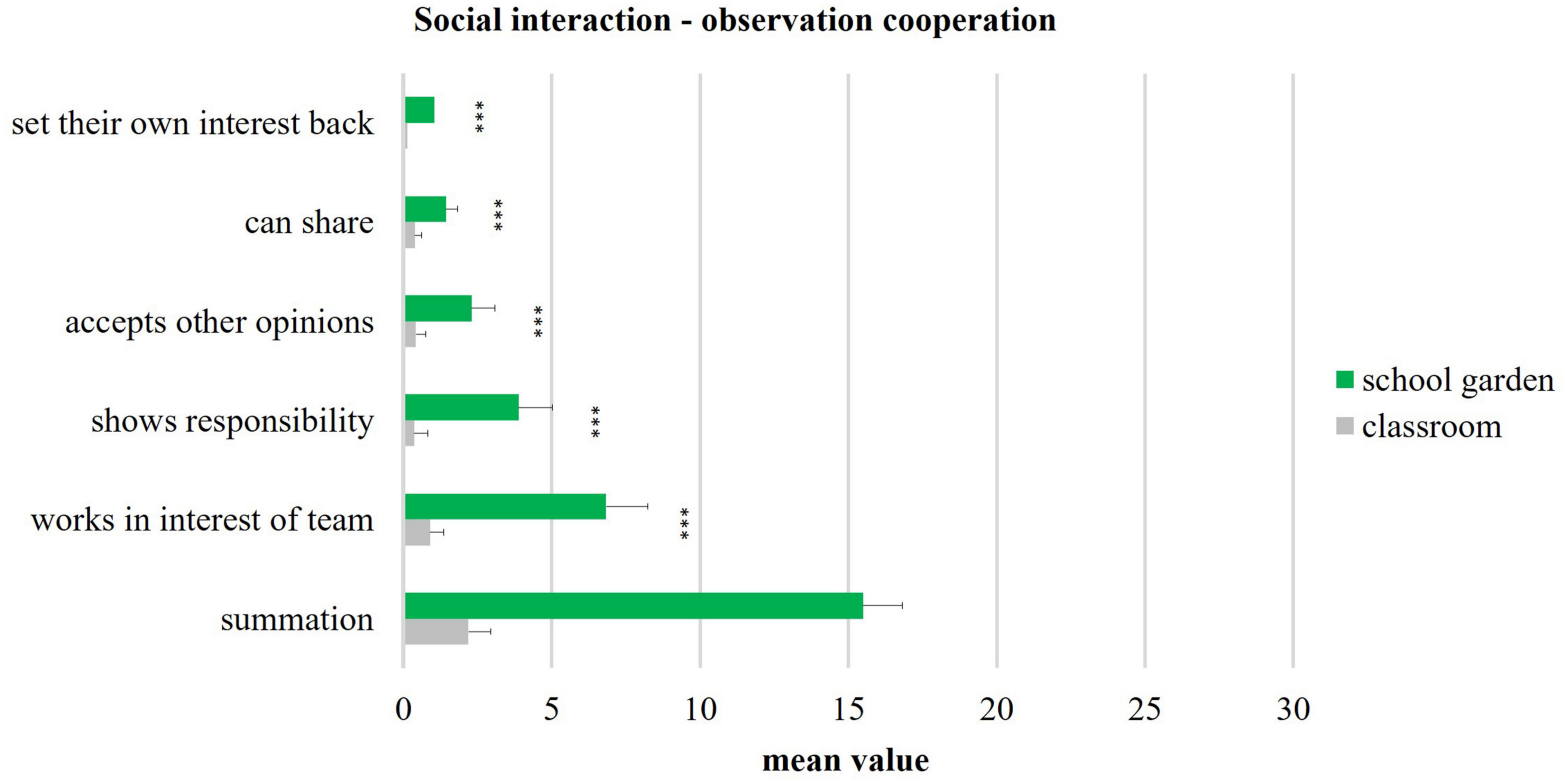
Social interaction - cooperation (school garden *n* = 82, classroom *n* = 68). Significance p different levels (*p < 0.05; **p < 0.01; ***p < 0.001).

The systematic behavioral observation attempted to show the frequency of social interactions between pupils during the lessons. The focus was put on the socially competent (prosocial) behavior of communication and cooperation. Because of the quantity of contacts, the type of interaction is crucial for the promotion of social skills. Over 150 observations by seven non-participating observers were held in the school garden (82) and in the classroom (68). Two sixth-grade classes were taught alternately in the school garden and in the classroom for 10 weeks (*n* = 53, 22 boys and 31 girls). The mean values of these two teaching locations are shown in Figures 8, 9. The comparison of school gardens (colored green) and classrooms (colored gray) shows small as well as large effects. Similar significant strong effects (according to Cohen, 1988, 1992) are evident for the three indicators of communication: “justifies criticism objectively” (*p* < 0.001; *d* = 1.46), “able to defend own point of view” (*p* < 0.001; *d* = 1.85), and “speaks clearly” (*p* < 0.001; *d* = 2.06).

The indicators of cooperation all show highly significant differences (*p* < 0.001) (Figure 9) and large effects (up value *d* = 1.727) in the comparison of school gardening vs. classroom. The mean values of classroom are all below the value of one and show a standard deviation with a maximum of 0.7. The mean values for school garden are between 1.4 and 6.8, with a standard deviation maximum of 1.4. For instance, the indicator “works in the interests of the team” with a mean value of 6.8 in the school garden has a very high effect of *d* = 5.386 (according to Cohen, 1988, 1992).

## Discussion

### Discussion of Methodology

It is important to stress that this study provides a description but cannot prove the causal relationships. Emotions and social interactions were investigated in the school garden and in the classroom.

The comparison of the results of both teaching locations serves to have a value for comparison, but cannot serve as a “control group” in the narrow sense. The school garden environment and the classroom environment differ not only in terms of location and specific circumstances, but also in the teacher's personal and social role during the lessons, as well as in the organization of lessons and the proportion of content in theory and practice. Several variables are in this investigation. This fact should be accepted in order to obtain data in a real school environment.

The investigation of behavioral observations is limited by human perception. This is why counting social interactions was done as the lowest level of description (Forgas and Frey, 1999) to document the observed interactions of the pupils in the school garden and in the classroom. The observers reported difficulties in observing the group of four pupils in the garden, when they moved away from each other. Negative behaviors were not measured in the observation because the observation would be too complex. Principally, in the sense of a mixed methods approach (Kuckartz, 2016), a qualitative investigation method (such as interviews) is recommendable, although the subjective distortion would be problematic.

The list of emotions in the emotion diary did not cause any irritations for the pupils, with the exception of the emotion “contempt.” This emotion does not seem to be known to the pupils. The acceptance of basic emotions is controversial, and there is still no clarification regarding this point. The criteria defined for basic emotions and the reducibility of secondary emotions to basic emotions are discussed (Siemer, 1999; Reisenzein, 2000). Likewise, the reflective view of oneself can also lead to deficiencies in emotional truthfulness. Immediate writing down of the emotions (at the time of perception) would be beneficial on one hand, but it is not practical and could even be counterproductive for the teaching process. Marginal memory errors due to the maximum delay of 60 min must therefore be accepted.

### Discussion of Results

The data from this study were collected as part of a repeat study with two classes of the sixth grade aged 11–12 years. The results of social interactions and perceived emotions were consistent with those of the first study (Pollin and Retzlaff-Fürst, 2018). As expected, it shows that the school garden offers more social opportunities and positive emotions than science lessons in the classroom.

The first question—What emotions were perceived by the pupils during the lessons in the classroom and in the school garden?—can be answered. Positive emotions, especially happiness and pride, were frequently indicated and the intensity of positive emotions was on a high level. The reasons given for the perception of emotions show a strong positive link to the nature experiences that were made in the garden. A wide range of emotions was covered in the emotion diary, but with regard to the valence of positive and negative emotions, there is an unbalanced ratio of three positive emotions to seven negative emotions. The reason for this is that there are more negative emotions in language usage. An imbalance in this valence can therefore also be recorded in the emotion diary and must be taken into account when interpreting the results.

The second question—Which social interactions of “socially competent behavior” can be observed in the school garden, in communication and cooperation?—can be answered. Social interactions during the school garden lessons are often observable, and cooperative behavior is particularly significant. External observation enables direct documentation, and there is no interruption of the lesson and no distortion due to memory deficits. A limitation of the study is that interrater reliability cannot be used to assess the quality of the observation. Each observer has only ever observed one group. For reasons of time, no further observer simulations could be carried out. However, observer feedback on handling the observer sheet was consistently positive. In order to obtain a future meaningful result of the observations, a supplementary method of substantiation might be possible. For example, video recordings of social interactions could be made.

Due to frequent social interactions and positive emotions, it can be assumed that garden-based learning promotes cooperative behavior. In reverse, this means that classroom lessons have a deficit in this field and should offer more opportunities for cooperation. As influencing factors for the positive effects, the stay in the green, the gardening, and the organization of lessons according to the basic needs of SDT can be considered. As observed in this study, pupils like to go to the garden, and based on their comments, pupils expressed particular joy and pride of their plants growing. Therefore, contact with nature is significant. This supports surveys, such as those by Blair (2009) and Passy et al. (2010), which emphasize the potential for the development of pupils through their work and being in school gardens. Other studies examine in particular the potential of staying in the countryside for health. These studies refer more generally to urban greenery and the environment of people of different ages (Hartig et al., 2014; Shanahan et al., 2016; Cox et al., 2017; Frumkin et al., 2017). Further studies on school gardens examine the effects of staying in green areas on health, such as eating behavior or mental health (Ozer, 2007; Leuven et al., 2018). The present results show that science lessons in the school garden, if oriented toward the basic needs of SDT, have effects on social behavior in secondary school children. Presumably, this also leads to better self-esteem of pupils and then it also has effects on health. Further studies are needed here. It should be examined whether these results also apply to higher classes—high school pupils. Only pupils of the sixth grade were examined in this study. The metastudy of Williams and Dixon (2013) also notes that examinations in higher grades (10–12th grade) are rare. It can be assumed that school garden lessons are valuable for inclusive classes, because work in the school garden gives particularly social and emotional positive stimuli.

## Data Availability Statement

The original contributions presented in the study are included in the article/Supplementary Material, further inquiries can be directed to the corresponding author.

## Ethics Statement

The investigation has been applied for and approved by the Ministry of Education, Science and Culture. Written informed consent to participate in this study was provided by the participants' legal guardian/next of kin.

## Author Contributions

All authors listed have made a substantial, direct and intellectual contribution to the work, and approved it for publication.

## Conflict of Interest

The authors declare that the research was conducted in the absence of any commercial or financial relationships that could be construed as a potential conflict of interest.
