# Supplementary material for: The School Garden: A Social and Emotional Place
Source: Front Psychol. 2021 Apr 22;12:567720. doi: 10.3389/fpsyg.2021.567720 (PMC8100502; doi:10.3389/fpsyg.2021.567720)
Supplement: Supplementary file 1 [file Data_Sheet_1.PDF]

Make your code name out of capital letters and numbers - please write clearly:

(1) the first two letters of your mother's first name **(2) the last two letters of your first name**
(3) the digits of your current house number.

Codename: ______________________________

Date: ______________________________

Gender: ☐ female ☐ male

ROstocker Schulgarten Akademie

Emotion-Diary

2 What were the following feelings in class?
Tick how strong you assess these feelings and briefly describe the associated situation.

Emotion	not felt	How intensive was your feeling?			Briefly describe in your own words what you did or experienced when you felt it.
		low	middle	high	
**rage/anger**	☐	☐	☐	☐	
**fear/anxiety**	☐	☐	☐	☐	
**wonder/surprise**	☐	☐	☐	☐	
**sadness**	☐	☐	☐	☐	
**disgust**	☐	☐	☐	☐	
**happiness**	☐	☐	☐	☐	
**contempt**	☐	☐	☐	☐	
**frustration**	☐	☐	☐	☐	
**pride**	☐	☐	☐	☐	
**embarrassment**	☐	☐	☐	☐	

Observation-Sheet – Social-interaction

Observation period:<br><br>Observation date:	Observed group:<br>Clothing/bibs<br>Special feature:
Time:<br><br>Sample number:	Observer:
	Location:<br>school garden or classroom

Observation takes place in 10-minute intervals - break of 5 minutes!

	**Indicators**	**Markedness (tally sheet)**				**Activity**
		Draw in to pupil symbol!	Draw in to pupil symbol!	Draw in to pupil symbol!	Draw in to pupil symbol!	
**Communication**	1) speaks clearly					
	2) justifies criticism objectively					
	3) keeps eye contact					
	4) able to defend own point of view					
	5) listen attentively					
**Cooperation**	6) can share					
	7) works in interest of team					
	8) set their own interest back					
	9) accepts other opinions					
	10) shows responsibility					

Notes

____

____

____

____
